# Physical Activity and Fundamental Motor Skill Performance of 5–10 Year Old Children in Three Different Playgrounds

**DOI:** 10.3390/ijerph15091896

**Published:** 2018-08-31

**Authors:** Jessie Adams, Jenny Veitch, Lisa Barnett

**Affiliations:** 1 School of Health and Social Development, Deakin University, Geelong 3220, Victoria, Australia; jessie.adams@deakin.edu.au; 2 Institute for Physical Activity and Nutrition (IPAN), School of Exercise and Nutrition Sciences, Deakin University, Geelong 3220, Australia; 3 Institute for Physical Activity and Nutrition (IPAN), School of Health and Social Development, Deakin University, Geelong 3220, Australia; lisa.barnett@deakin.edu.au

**Keywords:** physical activity, fundamental motor skills, playground, children, play, design

## Abstract

Playgrounds provide opportunities for children to engage in physical activity and develop their fundamental motor skills. The aim of this descriptive pilot study was to examine whether playground design facilitated different levels of physical activity and fundamental motor skills. Children aged 5 to 10 (*n* = 57) were recruited from three independent playgrounds located in Melbourne (Australia). Whilst playing, children wore accelerometers which measured time spent in physical activity and direct observations recorded fundamental motor skills and play equipment use. A general linear model with playground type as the predictor and adjusting for monitor wear-time identified whether mean time in physical activity was different for the three playgrounds. Frequencies and a one-way ANOVA assessed whether the observed mean number of fundamental motor skills varied between playgrounds. On average, 38.1% of time (12.0 min) was spent in moderate- vigorous-intensity physical activity. Children in the *traditional* playground (*n* = 16) engaged in more moderate-intensity physical activity (9.4 min) than children in the *adventure* playground (*n* = 21), (5.6 min) (*p* = 0.027). There were no significant associations with vigorous-intensity physical activity or fundamental motor skills between playgrounds. Children performed few fundamental motor skills but used a wider variety of equipment in the *contemporary* and *adventure* playgrounds. Playgrounds need to maximise opportunities for children to engage in physical activity and develop fundamental motor skills.

## 1. Introduction 

Regular participation in physical activity is important for overall health. It provides children with immediate social, mental and physical health benefits [1], such as reducing symptoms of depression, improving academic and cognitive performance, promoting healthy bone structure and skeletal health, supporting muscle growth and the development of the body’s vital organs, particularly the heart and lungs, improving health related fitness, muscular strength and, preventing children becoming overweight [2,3,4,5,6]. It is recommended by the Australian Government Department of Health [7] that children aged five to twelve years should participate in at least 60 min of moderate-to-vigorous intensity physical activity daily. Yet, only about one in five (19%) Australian children are meeting these guidelines on all days of the week [8]. 

Physical activity is influenced by factors at the individual, social and environmental level as per the social ecological model [9]. An important individual level correlate of children’s physical activity is adequate development of Fundamental Motor Skills (FMS) [10,11]. FMS are considered the building blocks (e.g., running and throwing) that lead to the development of specialised movement sequences which are refined and applied in physical activities throughout the lifespan [12]. Additionally, FMS development is associated with multiple health benefits, including; weight status, cardiorespiratory endurance, musculoskeletal fitness and muscular strength [13,14]. Despite the many benefits of adequate FMS, the most recent Australian representative survey found a high prevalence of primary-school children with low FMS [15]. It identified the side gallop as the most developed skill with 75% of boys and 80% of girls showing mastery, while the leap was the lowest skill level with 49% of boys and only 24% of girls showing mastery. 

A supportive built environment is important for children’s physical activity [9]. Playgrounds are identified at the community level of the social ecological model and form part of the built environment. They provide opportunities for children to engage in active play [16]. Community playgrounds are usually located within a public park, they are generally available in most neighbourhoods and free for public use [17]. Playgrounds have been identified as the place within parks where children expend the most energy [18]. Willenberg, et al. [19] suggested that it is important to better understand the influence of playground features and design on children’s physical activity levels. Many factors should be considered when designing playgrounds including the amount of available space, configuration and safety of equipment, degree of challenge, and novelty [17,20]. To maximize public health impact, it is important for playground equipment to meet the needs of children with diverse motor skills across age groups. In addition, playground equipment should be designed to encourage physical activity [21,22,23]. 

Limited research has examined associations between specific community playground equipment and design and physical activity among children. A qualitative study that examined the perceptions of 78 parents of primary school-aged children regarding factors influencing where children play found that playgrounds are typically designed for young children with older children preferring more adventurous and challenging equipment [22]. Willenberg, Ashbolt, Holland, Gibbs, MacDougall, Garrard, Green and Waters [19] studied children aged 9–11 years from 23 Melbourne schools using the System for Observing Play and Leisure Activity in Youth [24] and identified that a higher availability of loose equipment, such as balls, encourages vigorous activity in a playground. In addition, Lindberg and Schipperijn [25] identified that children (under 13 years) were most active on standard playground equipment such as swings and climbing frames. In summary, it appears playgrounds are designed with younger children in mind and that loose equipment as well as standard fixed equipment may encourage physical activity.

Limited research has examined the role of playgrounds in developing children’s FMS or on the level of FMS needed for children to engage in playground activity. A study among Italian pre-school children found that a combination of structured and unstructured play was important in the development of FMS [26]. Aside from that study, it is not known what specific playground design encourages a wide use of FMS. Once play equipment is installed it is generally there for a long time and is costly to purchase, install and maintain. It is therefore important to understand what equipment best meets children’s needs, optimises physical activity and helps develop FMS. It is also important to better understand parent views on what features may encourage children to be more active at the playground [22].

The aim of this pilot study was to objectively measure (using accelerometers) the activity levels of children aged 5–10 years in a variety of community playground settings and to observe the FMS and equipment used during play. The relationship between different playground designs and children’s physical activity and FMS engagement was also examined. Parental perceptions on reasons their child visits the park and park features that may encourage their child to be more active were also explored. It was hypothesised that: (i) children would participate in more physical activity at playgrounds with a greater range of equipment; (ii) children would perform a wider range of FMS in playgrounds with a greater range of equipment, and (iii) children would play on a wider range of equipment at playgrounds providing more diverse equipment. 

## 2. Methods 

Low-risk ethical approval was granted by the University’s Research Ethics Committee, reference HEAG-H 37_2017.

### 2.1. Participant Recruitment 

Children were recruited at each playground on the day of data collection using convenience sampling. A researcher (Author 1) approached the child’s parent(s), explained the study and assessed if the child met the selection criteria: child aged 5–10 years with parent or legal guardian present to consent, without a physical condition that would affect their ability to play, be at the playground for the next 20 min to allow for observations to be completed, and no previous participation in the study.

In total, 73 parents were approached and had a child that met the selection criteria, with 61 consenting to their child’s participation (83.6% consent rate). The main reasons for declining participation included parents not wanting to stay at the playground for another 20 min, or not wanting their child to wear an accelerometer. 

Of the 57 children included in final analysis; 16 (eight girls, eight boys) were recruited at the traditional playground (28.7%), 20 (eight girls, 12 boys) at the contemporary playground (35.1%) and 21 (12 girls, nine boys) at the adventure playground (36.8%). The mean age of participants was 6.9 years (SD: 1.5) and was similar between the playgrounds; traditional (7.5 years, SD: 1.8), contemporary (6.5 years, SD: 1.2) and adventure (6.9 years, SD: 1.6). Overall, there was an equal mix of boys (*n* = 29, 50.9%) and girls (*n* = 28, 49.1%). 

### 2.2. Design

Three playgrounds located within a 10-km radius in metropolitan Melbourne (Australia) were selected based on their varying design, equipment, size and scope for physical activity (Table S1). The traditional playground was the smallest of the three and consisted 27 different play items including; five main metal structures, six slides, four ladders, a fireman’s pole, swings, a see-saw and rockers. The contemporary playground was unique as it had modern and traditional styled equipment spread over a large area. It consisted 37 pieces of equipment, including; monkey bars, five slides, a flying fox, castle and mound. The adventure playground was a large modern adventure playground centred around a large ramp with 30 pieces of equipment, including; a bird-nest swing, rock climbing wall, climbing nets and a spinner chair (images shown in Figure S2). 

Data collection occurred during July and August 2017 (winter), on weekends and weekdays during the school holidays. Weather was similar on all days of data collection (average 14 degrees Celsius and not raining). All three playgrounds were visited on each study day (to minimise effect of weather on play in different playgrounds) for approximately two hours and each playground was visited five to seven times to recruit participants (adventure playground = five visits, contemporary playground = six visits, traditional playground = seven visits). 

### 2.3. Measurements 

GT1M ActiGraph accelerometers [27] were worn by the children on the right hip to objectively assess activity levels (light, moderate and vigorous intensity) while children were playing at the playground. The accelerometers were placed on the children immediately prior to observations (see below) and were removed when the children left the playground. 

Observation scans of the selected child were conducted by Author 1 using a modified version of the System for Observing Fitness Instruction Time (SOFIT) [28]. Observers only observed one child at a time. Individual observation charts were developed for each playground to allow the different equipment in each playground to be listed on the observation form along with 19 FMS (see Figure S2). The FMS list was grouped into categories: locomotor skills (walk, run, crawl, leap, jump, skip/hop and dodge/slide); object control skills (kick, catch, throw, strike and roll); body management skills (balance, climb, hang and pull) and sedentary/low active actions (stand, sit, lie) [29,30]. Each observation scan recorded the FMS being performed by the child, the piece of equipment the child was playing on and whether the child was playing alone or with others. Observations for each child were completed every 20 s for a total of 15 min using the SOFIT instrument, resulting in a total of 45 observations per child. Each child was only observed once. Observations began 2–3 min after the accelerometer was attached to the child to account for reactivity bias. One observer completed all observations, with this observer having completed prior training using SOFIT training videos and in-field training to ensure the observations were accurate. Additionally, pilot testing occurred at the park on one day in which observations were conducted, first collaboratively and then independently, with a researcher with expertise in conducting direct observations in playgrounds. Inter-rater reliability was assessed to ensure reliability and the result between the two researchers was 90% based on percent agreement. 

Pen and paper surveys regarding playground use, developed from a survey previously used by Veitch, et al. [31], were completed by the parents while the playground observations were being conducted. The survey included the following items: “did you come to the playground from your home (yes, no)?”, “how do you usually get to this playground (car, walk, public transport, bike/cycle/scooter, other)?”, “why do you come to this playground (open ended)?”, and “what features of the playground do you think encourages your child to be most active (open ended)?”. 

### 2.4. Data Analysis

Of the 61 consenting children, participants without complete data for all 45 observation scans were removed (*n* = 4) leaving a final sample of 57 children. Accelerometer data was initially uploaded onto the ActiLife (v6.13.3) software (ActiGraoh, Pensacola, FL, USA). During initialisation the epoch rate was set to 15 s due to the short-burst nature of children’s play [32]. An Excel macro was then used to produce the average minutes of light, moderate and vigorous intensity physical activity per child based on Evenson’s cut-points [33]. Following this, IBM SPSS Statistics v 24 (IBM, Armonk, NY, USA) was used to calculate frequencies and descriptive statistics. A general linear model with mean-minutes of physical activity (either moderate or vigorous) as the outcome, playground type as the predictor (using the adventure playground as the reference) and adjusting for monitor wear-time was used to identify whether time in physical activity was different for children playing at the three playgrounds. Bonferroni post-hoc tests were used to identify any significant pairwise comparisons between the three playgrounds.

Observational data was coded (according to the number codes written on the observation form see Figure S2) and entered into an Excel worksheet and then transferred to SPSS for analysis. Frequencies and demographic information was calculated and then one-way ANOVAs were conducted to assess whether the mean number of FMS observations varied between playgrounds. As equipment in each playground was different, no statistical analysis to examine equipment usage across playgrounds could be performed. 

## 3. Results

### 3.1. Physical Activity 

On average, children wore the accelerometers for 31.5 min (traditional: 33.1 min, SD: 15.3); contemporary: 34.5 min (SD: 18.3); adventure: 27.3 min (SD: 8.7). As shown in Table 1, 38.1% of time (12.0 min) was spent in moderate-vigorous-intensity physical activity (MVPA). Children in the traditional playground spent 42% (13.9 min, SD: 3.5) of their time in MVPA, the highest of all three playgrounds. Time in vigorous activity was similar between the adventure (17.6%, 4.8 min, SD: 3.4), traditional (13.6%, 4.5 min, SD: 3.6) and contemporary playground (11.6%, 4.0 min, SD: 2.4). 

### 3.2. Association between Playground Type and Physical Activity 

The general linear model identified a significant association (*p* = 0.03) in time spent in moderate intensity physical activity according to the playground. This model explained a total of 51% adjusted variance in moderate activity (Table 2). According to post-hoc tests (not shown in the table), children spent significantly more time in moderate intensity activity at the traditional playground compared to the adventure playground, 2.72 mean difference, *p* = 0.023. There were no statistically significant associations in children’s vigorous-intensity physical activity across the three playgrounds. There were also no associations with MVPA (data not shown).

### 3.3. Observation of Fundamental Movement Skills

A one-way ANOVA determined there were no statistically significant associations in the total number of different FMS observed across the three playgrounds (F (2,54) = 0.2, *p* = 0.855). The most common behaviours performed were sedentary/low active (52.9% of all observations), in particular standing (26.3%) and sitting (24.5%) (Table 3). The most frequently performed FMS were locomotor skills (31.3%), specifically walking (18.3%) and running (11.3%). Body management skills (15.2%), in particular climbing (12.3%), were also observed at all three playgrounds, whereas object control skills such as catching and throwing were rarely observed (0.0%, 0.2%).

### 3.4. Playing Alone or with Others 

Children were observed playing or engaging with others in 84.6% of all observations. This was most common at the traditional playground where children were observed playing with others almost all of the time (99.2%), compared to most of the time (81.1% of observations) at the *contemporary* playground and around three quarters of the time (76.7%) at the adventure playground. ‘Others’, were typically siblings, parents or peers. 

### 3.5. Equipment Use Across the Playgrounds 

At the traditional playground, playing on ‘no equipment’ was observed most frequently (28% of all observations). The most commonly used equipment were the slides (20.0%), oval (19.0%) and platform structures (10.8%). At the contemporary playground, ‘no equipment’ was also observed most frequently (19.0%) and the most commonly used equipment included the platforms (9.4%), flying foxes (8.8%), mound (7.1%) and the spin roundabout (6.9%). The most commonly used equipment at the adventure playground were the climbing nets (21.2%), swings (10.5%), spinner chair (10.2%) and skate park (9.2%). The adventure and contemporary playgrounds had more equipment than the traditional playground, so recordings were more dispersed across equipment. 

### 3.6. Parent Survey 

The majority of participants came to the playground from home (*n* = 47, 87.0%). Overall, the car was the most common method of transport to the playground (*n* = 38, 55.1%); however, there were slight variations between the three playgrounds. Walking was the main method of transport to the traditional playground (47.8%); while the car was most commonly used for the contemporary (50.0%) and adventure playgrounds (78.3%). 

Parents provided multiple reasons for visiting the three playgrounds. Overall, the most common responses were ‘convenient location’ (*n* = 37) and ‘design’ (*n* = 36). Other less frequently reported responses were ‘size’ (*n* = 12), ‘safety’ (*n* = 12), ‘fun/social’ (*n* = 12) and ‘exercise’ (*n* = 11). There were slight variations in responses between playgrounds. ‘Convenient location’ was the most commonly reported reason parents took their children to the traditional playground (*n* = 20) whereas ‘design’ was commonly reported by parents at both the contemporary (*n* = 11) and adventure playgrounds (*n* = 22). Additionally, ‘exercise’; was identified as a popular reason to attend the contemporary playground (*n* = 7) and safety for the adventure playground (*n* = 9). 

Parents most commonly reported that the play equipment was an aspect of a playground that encouraged physical activity (*n* = 39) followed by, ‘design’ (*n* = 25) and ‘climbing features’ (*n* = 23). At the traditional playground, parents highlighted that the ‘design’ (*n* = 9) and ‘climbing features’ (*n* = 8) encouraged their child to be active. The ‘flying fox’ (*n* = 9), ‘space’ (*n* = 8) and ‘climbing equipment’ (*n* = 8) were acknowledged as important components of the contemporary playground, whereas parents at the adventure playground reported that the overall ‘design’ (*n* = 11) and the choice of ‘different equipment’ (*n* = 10) encouraged activity play. 

## 4. Discussion

The aim of this study was to investigate the physical activity levels of children aged 5–10 years in a variety of community playground settings and to observe the FMS and equipment used during playground play. The results show that playground play can contribute significantly to children’s daily physical activity needs. On average, children wore the accelerometer for 31 min and of this, engaged in 12 min of MVPA which is nearly a quarter of the recommended 60 min of daily MVPA [7]. As most children were only observed for a short period of their time at the playground, it can be acknowledged that time spent at the playground can significantly contribute to children’s daily physical activity requirements. 

It was hypothesised that children would participate in higher amounts of physical activity at playgrounds with a greater range of equipment (i.e., the adventure and contemporary playgrounds). Mixed results were found in relation to activity levels. Children spent more time in objectively measured moderate intensity physical activity at the traditional than at the adventure playground. This finding does not support the original hypothesis as the traditional playground had the smallest range of equipment. Anecdotally, however, it was observed that children at the traditional playground commonly engaged in active running games such as ‘chasey’, to keep themselves entertained-possibly due to the lack of equipment offered and this may have contributed to their higher activity levels. 

Salvy, et al. [34] identified that in a variety of settings and contexts, children engaged in less intense activity when alone and more intense activity when with peers. In the current study, children at the *traditional* playground were observed playing with others almost exclusively (99%) of the time compared to 81% at the contemporary and 77% at the adventure playgrounds. This greater participation in interactive play may have also encouraged more active play. Furthermore, it was observed anecdotally that parents at the traditional playground were more engaged in their children’s play compared to parents at the adventure playground who tended to observe from a distance. Engaged parents have been shown to encourage children to play by creating a fun atmosphere around play and physical activity [35]. This suggests that playing with others may positively influence physical activity levels [36]. 

There was no statistically significant associations with vigorous intensity activity between the three playgrounds. Dyment, et al. [37] reported children in kindergarten to grade 8 were more likely to engage in vigorous physical activity on manufactured playground equipment (including slides, monkey bars and swings) compared to other areas including open green spaces. That study also identified that the presence of open green spaces at playgrounds encouraged moderate intensity physical activity [37] which is consistent with our findings from the traditional playground, where the third most popular amenity used was the oval. Children in the contemporary playground spent the least amount of time in vigorous physical activity. This is inconsistent with our hypothesis as the contemporary playground was a large playground with equipment spread apart requiring children to move substantial distances to play on different equipment [38]. A previous study by Farley, et al. [39] supports this unexpected finding. They identified by observation, that children in grades two to eight were more active in high density areas and suggested that playgrounds do not need to be spread out to encourage physical activity in school-aged children. At the contemporary playground, children were most frequently observed standing on the platforms (usually waiting for a turn to use the monkey bars or flying fox), using the flying fox and sitting on the spin roundabout. These commonly used pieces of equipment did not appear to encourage vigorous, or even moderate intensity physical activity (aside from hanging when on the flying fox and monkey bars). 

Overall, sedentary/low active behaviours were the most common behaviour observed and children spent the majority of time on ‘no equipment’. Previous research among preschool children found those with low motor competence tended to spend more time in sedentary activities on and around the playground compared to children with high motor competence [40]. Considering many Australian primary-school children do not master FMS [15] it is important to manufacture built environments which provide opportunities for children to practice FMS, and speculatively (even though FMS competence was not measured in this study), playgrounds could be one setting to support this. Review studies examining pre-school and school play spaces have found mixed evidence for associations between school yard playgrounds and increased physical activity [41,42]. 

In fact, our second hypothesis was that children would perform a wider range of FMS in playgrounds with a diverse range of play equipment. However, there were no statistically significant associations between the FMS observed at the three playgrounds. It is possible that the low FMS mastery among Australian children could be influenced by the lack of FMS required to play in playgrounds [43]. As Tortella, et al. [44] identified, free play alone is not enough to develop children’s FMS; a mixture of structured and unstructured play as well as specifically targeted play equipment may be necessary to encourage physical activity and FMS development. Furthermore, instruction is considered very important for adequate FMS development, with a systematic review identifying that control groups engaged in free play did not improve in their FMS compared to intervention groups with instruction [45]. This may be particularly important for object control skills and other skills such as balance that were rarely observed across the playgrounds.

Locomotor skills such as walking and jogging were observed more often in the *contemporary* playground. This was anticipated due to the equipment being spread out over a large area requiring children to use locomotor skills to move around. Conversely, locomotor skills were observed less frequently at the adventure playground, which may be due to the equipment primarily being linked off a large walkway meaning children required differing FMS (such as balance) to move around the various equipment. Overall, climbing was identified as the second most observed FMS at all three playgrounds and ‘climbing nets’ were the most used piece of equipment at the *adventure* playground. This is important, as apart from trees, there are few places children can practice their climbing and hanging skills and develop upper-body strength [46]. Recently, Australian legislation has prompted the removal of playground equipment that is perceived dangerous, such as monkey bars and swings [47]. The removal of this equipment could negatively influence the development of climbing and hanging skills as children will have reduced opportunities to develop their upper-body strength, which is an important outcome. Developing such skills allows children to build confidence, strength and develop risk-taking assessment [47] which may encourage participation in other forms of physical activity, such as competitive sports. The removal of adventurous and challenging equipment may also discourage children from visiting playgrounds as we know from previous research that children desire physically challenging and adventurous play equipment, and this may lead to even lower levels of physical activity and FMS performance [22,23]. Park planners should consider FMS when designing playgrounds and incorporate equipment to provide opportunities for children to enhance particular FMS such as object control and balance [43]. For example, more creative designs could be considered such as a ball on a string that children could throw or bat to each other. 

Our third hypothesis was that children would play on a wider range of equipment at playgrounds providing more diverse equipment. More equipment was present at the adventure and contemporary playgrounds, compared to the traditional playground, and it was evident that children were using the wider range of equipment at these playgrounds. Children in the current study spent considerable time on the standard playground equipment, such as ‘swings’ and ‘climbing frames’. This was particularly evident in the traditional playground, with 20% of observations on the ‘slides’ alone. Additionally, over 30% of the observations at the adventure playground were on the ‘climbing nets’ and ‘swings’. Moreover, when parents were asked what features of the playgrounds encouraged their child’s physical activity, the most common responses were ‘swings’, ‘slides’ and ‘climbing’ facilities. This highlights the importance and value of traditional equipment and suggests it is still well-used by children. Some of the standard equipment had a modern twist, for example the ‘climbing nets’ in the adventure playground comprised a series of various spiders’ nets, and it is possible that these variations create excitement and interest for children. Lindberg and Schipperijn [25] used SOPARC to examine the use of urban green spaces and found the use of regular playground equipment such as ‘swings’ and ‘slides’ were associated with increased physical activity among children [25]. This is aligned with the results from the current study as activity levels were higher in the traditional playground where standard equipment was most present. Playgrounds need to cater to a vast range of age and development levels; therefore, it is important for future research to better understand what equipment is preferred by children of different ages to ensure equipment meets children’s needs, encourages them to visit and be active and develop FMS during their visit. 

Parents reported that ‘convenient location’ was the main reason for attending the traditional playground compared to ‘design’ in both the contemporary and adventure playgrounds. Furthermore, the most common transport method to the adventure playground was ‘car’, in contrast to ‘walking’ to the traditional playground. This suggests that parents are willing to travel further to visit a playground with more diverse equipment. 

### Strengths and Limitations 

The use of multiple methods to examine children’s behaviour in various playgrounds is one strength of this study. Using objective measures to assess physical activity and conducting direct observations is a strength compared to using child-report or parent-proxy instruments [48]. Furthermore, the one researcher completed all observations, so the results are not influenced by inter-rater reliability, although lack of interrater comparison can also be viewed as a limitation when developing a new checklist. The observation and survey were modified from previously used instruments [49] and the completion of pilot testing prior to undertaking any data collection assured the instruments and protocol were appropriate for the research methods [50], although as this is the first time this checklist has been used this may also be considered a limitation. 

Whilst, recruitment of participants in the contemporary and adventure playground was achieved with ease, it was a challenge at the traditional playground as few children visited the playground. Future research would benefit from investigating playgrounds varying in size, features, amenities, and location. A larger sample size would have also enabled the statistical models to be adjusted by age and sex. 

A further possible limitation of the observational cross-sectional data was that the ‘no equipment’ observations included participants in transit between using various pieces of equipment. In addition, at the traditional playground, it was easy to observe children from the one vantage point, however, in the two larger playgrounds this was more challenging and it was necessary to move around to ensure participants could be seen clearly whilst also trying to remain as inconspicuous as possible so as not to influence the participant’s play [51]. 

It is also worth noting that data was collected in winter months which may have affected play behavior, however, there is no reason to suggest this would affect behaviour across the playgrounds differently. It is also possible that parents may have provided socially correct responses to the survey questions. Further, it would have been useful to have collected additional demographic information from children and their parents (e.g., race, ethnicity, income) in order to better describe the sample. 

Finally, it is recognised that all three playgrounds were located within close proximity to each other and were in a neighbourhood of similar socio-economic status; however, this may not be seen as a limitation, as the study aimed to examine children’s engagement with the playground design rather than comparing differences across socio-economic status areas. It is understood, however, that playgrounds in lower socio-economic areas may have different facilities to the playgrounds included in the current study [52] and that children in such areas may have less access to resources which promote FMS development. 

## 5. Conclusions

This pilot study showed that playground play has the potential to significantly contribute to children’s daily physical activity needs, as on average, 38% of time was spent in MVPA. Contrary to expectations, children in the traditional playground spent the most time in moderate intensity physical activity. From a public health standpoint, further research is required to better understand activity levels of children using playgrounds of varying size and design. There were no significant differences between the FMS performed at the three playgrounds, although, walking, running and climbing were the most frequently observed skills. As hypothesised, children at the two larger playgrounds, spent more time playing on a greater variety of equipment than children at the traditional playground. Overall, these findings suggest that playground play can contribute significantly to children’s physical activity requirements, although it appears to involve a limited number of FMS. The results of this research can inform future studies on this topic and assist future planning of playgrounds to optimise playground effects on physical activity and enhance FMS among our children. Future research would benefit from investigating playgrounds of varying size, features, amenities, and location to ensure playgrounds meet the needs of children of all ages and developmental levels.

## Figures and Tables

**Table 1:** Time spent in physical activity at each playground.

Physical Activity Level	Overall		Traditional Playground		Contemporary Playground		Adventure Playground	
	%	Mean Min (SD)	%	Mean Min (SD)	%	Mean Min (SD)	%	Mean Min (SD)
Light	48.9	15.4 (8.5)	51.7	17.1 (9.3)	49.6	17.1 (10.1)	27.3	12.4 (5.2)
Moderate (M)	24.1	7.6 (4.1)	8.4	9.4 (4.5)	23.8	8.2 (4.3)	20.5	5.6 (2.8)
Vigorous (V)	4.0	4.4 (3.1)	13.6	4.5 (3.6)	11.6	4.0 (2.4)	17.6	4.8 (3.4)
MVPA	38.1	12.0 (2.3)	42.0	13.9 (3.5)	35.4	12.2. (3.0)	38.1	10.4 (0.6)

Table 1 shows the average amount of time participants spent in light, moderate, vigorous and MVPA at each playground measured by accelerometry. Note: % does not add up to 100% as participants also spent time sedentary.

**Table 2:** Associations between playground type and children’s physical activity.

Parameter	*B*	*p*	95% Confidence Interval	
			Lower Bound	Upper Bound
Outcome—Moderate intensity physical activity				
Intercept	0.71	0.469	−1.25	2.67
Wear-time	0.18	0.000	0.13	0.23
Traditional	2.72	0.008	0.76	4.68
Contemporary	1.34	0.157	−0.53	3.20
Adventure	0 ^a^	-	-	-
Adjusted R Squared = 0.506				
Outcome—Vigorous intensity physical activity				
Intercept	1.75	0.061	−0.08	3.58
Wear-time	0.11	0.000	0.06	0.16
Traditional	−1.01	0.274	−2.85	0.83
Contemporary	−1.68	0.059	−3.43	0.06
Adventure	0 ^a^	-	-	-
Adjusted R Squared = 0.240				

Table 2 shows the general linear model of physical activity and the three playgrounds. ^a^ The adventure playground was the comparator playground. *B* = regression coefficient. *p* = statistical significance set at <0.05.

**Table 3:** FMS (Fundamental Motor Skills) observed at each playground.

FMS (Fundamental Motor Skills)	Overall %	Traditional Playground *N* (%)	Contemporary Playground *N* (%)	Adventure Playground *N* (%)
Sedentary/low active	52.9	394 (54.8)	438 (48.7)	524 (55.4)
Stand	26.3	223 (31.0)	240 (26.7)	211 (22.3)
Sit	24.5	151 (21.0)	178 (19.8)	299 (31.6)
Lie	2.1	20 (2.8)	20 (2.2)	14 (1.5)
Locomotor skills	31.3	236 (32.9)	321 (35.7)	245 (26.0)
Walk	18.3	143 (19.9)	194 (21.6)	132 (14.0)
Run	11.3	82 (11.4)	108 (12.0)	100 (10.6)
Jump	0.9	5 (0.7)	9 (1.0)	8 (0.9)
Crawl	0.6	4 (0.6)	7 (0.8)	5 (0.5)
Dodge/slide	0.2	2 (0.3)	3 (0.3)	0
Leap	0	0	0	0
Skip/hop	0	0	0	0
Body management skills	15.2	84 (11.7)	134 (14.9)	172 (18.2)
Climb	12.3	64 (8.9)	100 (11.1)	151 (16.0)
Hang	2.5	18 (2.5)	32 (3.6)	14 (1.5)
Balance	0.4	2 (0.3)	2 (0.2)	7 (0.7)
Pull	0	0	0	0
Object-control skills	0.6	6 (0.7)	7 (0.8)	4 (0.4)
Kick	0.4	5 (0.7)	6 (0.7)	0
Throw	0.2	1 (0.01)	0	4 (0.4)
Catch	0.0	0	1 (0.1)	0
Strike	0	0	0	0
Roll	0	0	0	0

Table 3 shows the number of FMS observed at each playground. Participants were observed and their FMS were recorded every 20 s for a 15 min period.

## Supplementary Materials

The following are available online at http://www.mdpi.com/1660-4601/15/9/1896/s1, Table S1: Comparison of equipment between playgrounds, Figure S1: Images of playgrounds (a): Traditional playground; (b): Contemporary playground; (c): Adventure playground, Figure S2: Observation chart (Observation chart developed for the contemporary playground. For each scan, the corresponding FMS number was written in the box matching the piece of equipment the child was playing on).

## References

[1] Boreham C., Riddoch C. (2010). The physical activity, fitness and health of children. J. Sports Sci..

[2] Gunter K.B., Almstedt H.C., Janz K.F. (2012). Physical activity in childhood may be the key to optimizing lifespan skeletal health. Exerc. Sport. Sci. Rev..

[3] Saris W., Blair S.N., van Baak M. (2003). How much physical activity is enough to prevent unhealthy weight gain? Outcome of the IASO 1st Stock Conference and consensus statement. Obes. Res..

[4] Boreham C., McKay H. (2011). Physical activity in childhood and bone health. Br. J. Sports Med..

[5] Fedewa A., Ahn S. (2011). The effects of physical activity and physical fitness on children’s acheivement and cognitive outcomes: A meta-analysis. Res. Q. Exerc. Sport..

[6] Hoare E., Skouteris H., Fuller-Tyszkiewicz M., Millar L., Allender S. (2013). Associations between obesogenic risk factors and depression among adolescents: A systematic review. Obes. Res. Clin. Pract..

[7] Australian Government Department of Health (2014). Australia’s Physical Activity and Sedentary Behaviour Guidelines.

[8] Australian Bureau of Statistics Australian Health Survey: Physical Activity, 2011-12; 5–17 Year Olds. http://www.abs.gov.au/ausstats/abs@.nsf/lookup/462FBA87B642FCA4CA257BAC0015F3CE?opendocument.

[9] Sallis J.F., Owen N., Fisher E.B. (2008). Ecological Models of Health Behaviour. Heath Behaviour and Health Education: Theory, Research, and Practice.

[10] Logan S.W., Webster E.K., Getchell N., Pfeiffer K.A., Robinson L.E. (2015). Relationship between fundamental motor skill competence and physical activity during childhood and adolescence: A systematic review. Kinesiol. Rev..

[11] Robinson L.E., Stodden D.F., Barnett L.M., Lopes V.P., Logan S.W., Rodrigues L.P., D’Hondt E. (2015). Motor competence and its effect on positive developmental trajectories of health. Sports Med..

[12] Clark J.E., Metcalfe J.S. (2002). The mountain of motor development: A metaphor. Motor Development: Research and Reviews.

[13] Cattuzzo M.T., Henrique R.S., Re A.H.N., Oliveira I.S., Melo B.M., Moura M.S. (2016). Motor competence and health related physical fitness in youth: A systematic review. J. Sci. Med. Sport..

[14] Stodden D.F., Goodway J.D., Langendorfer S.J., Roberton M.A., Rudisill M.E., Garcia C., Garcia L.E. (2008). A developmental perspective on the role of motor skill competence in physical activity: An emergent relationship. Quest.

[15] NSW Government (2015). NSW School Physical Activity and Nutrition Survey (SPANS) 2015.

[16] Berg S. (2015). Children’s activity levels in different playground environments: An observational study in four canadian preschools. Early Child. Educ. J..

[17] Hyndman B., Telford A. (2015). Should educators be ‘wrapping school playgrounds in cotton wool’ to encourage physical activity? Exploring primary and secondary students’ voices from the school playground. Aust. J. Teach. Educ..

[18] Besenyi G.M., Kaczynski A.T., Wilhelm Stanis S.A., Vaughan K.B. (2013). Demographic variations in observed energy expenditure across park activity areas. Prev. Med..

[19] Willenberg L.J., Ashbolt R., Holland D., Gibbs L., MacDougall C., Garrard J., Green J.B., Waters E. (2010). Increasing school playground physical activity: A mixed methods study combining environmental measures and children’s perspectives. J. Sci. Med. Sport.

[20] Barbour A.C. (1999). The impact of playground design on the play behaviors of children with differing levels of physical competence. Early Child. Res. Q..

[21] Clements R. (2004). An investigation of the status of outdoor play. Cont. Issues Early Child..

[22] Veitch J., Bagley S., Ball K., Salmon J. (2006). Where do children usually play? A qualitative study of parents’ perceptions of influences on children’s active free-play. Health Place.

[23] Veitch J., Salmon J., Ball K. (2007). Children’s perceptions of the use of public open spaces for active free-play. Child. Geogr..

[24] McKenzie T., Marshall S.J., Sallis J.F., Conway T.L. (2000). Leisure-time physical activity in school environments: An observational study using soplay. Prev. Med..

[25] Lindberg M., Schipperijn J. (2015). Active use of urban park facilities—Expectations versus reality. Urban For. Urban. Green.

[26] Tortella P., Fumagalli G., Loras H., Haga M., Sigmundsson H. (2014). Exploring the effects and specificity of playground activities on motor skills in 5 years old children. Sci. Sports.

[27] Pober D.M., Staudenmayer J., Raphael C., Freedson P.S. (2006). Development of novel techniques to classify physical activity mode using accelerometers. Med. Sci. Sports Exerc..

[28] McKenzie T.L., Sallis J.F., Nader P.R. (1992). SOFIT: System for observing fitness instruction time. J. Teach. Phys. Educ..

[29] Lubans D.R., Morgan P.J., Cliff D.P., Barnett L.M., Okely A.D. (2010). Fundamental movement skills in children and adolescents. Sports Med..

[30] Department of Education WA Department of Education, Western Australia 2013. http://det.wa.edu.au/stepsresources/detcms/navigation/fundamental-movement-skills/.

[31] Veitch J., Salmon J., Carver A., Timperio A., Crawford D., Fletcher E., Giles-Corti B. (2014). A natural experiment to examine the impact of park renewal on park-use and park-based physical activity in a disadvantaged neighbourhood: The REVAMP study methods. BMC Public Health.

[32] Pate R.R., Almeida M.J., McIver K.L., Pfeiffer K.A., Dowda M. (2006). Validation and calibration of an accelerometer in preschool children. Obesity.

[33] Evenson K.R., Catellier D.J., Gill K., Ondrak K.S., McMurray R.G. (2008). Calibration of two objective measures of physical activity for children. J. Sport Sci..

[34] Salvy S., Bowker J., Roemmich J., Romero N., Kieffer E., Paluch R., Epstein L.H. (2008). Peer influence on children’s physical activity: An experience sampling study. J. Pediatr. Psychol..

[35] Gustafson S., Rhodes R. (2006). Parental correlates oh physical activity in children and early adolescents. Sports Med..

[36] Salvy S., Roemmich J., Bowker J., Romero N., Stadler P., Epstein L. (2009). Effect of peers and friends on youth physical activity and motivation to be phyiscally active. J. Pediatr. Psychol..

[37] Dyment J.E., Bell A.C., Lucas A.J. (2009). The relationship between school ground design and intensity of physical activity. J. Child. Geogr..

[38] Dowda M., Brown W.H., McIver K.L., Pfeiffer K.A., O’Neill J.R., Addy C.L., Pate R.R. (2009). Policies and characteristics of the preschool environment and physical activity of young children. Pediatrics.

[39] Farley T.A., Meriwether R.A., Baker E.T., Rice J.C., Webber L.S. (2008). Where do the children play? The influence of playground equipment on physical activity of children in free play. J. Phys. Act. Health.

[40] Tsuda E., Goodway J.D., Famelia R., Brian A.S., Collins M.E. (2016). The relationship between motor competence and sedentary behaviour on the preschool playground. Res. Q. Exerc. Sport.

[41] Escalante Y., Garcia-Hermoso A., Backx K., Saavedra J.M. (2014). Playground designs to increase physical activity levels during school recess: A systematic review. Health Educ. Behav..

[42] Broekhuizen K., Scholten A.M., Vries S.I. (2014). The value of (pre)school playgrounds for children’s physical activity level: A systematic review. Int J. Behav. Nutr. Phys. Act..

[43] NSW Government (2011). NSW Schools Physical Activity and Nutrition Survey (SPANS) Health.

[44] Tortella P., Haga M., Loras H., Sigmundsson H., Fumagalli G. (2016). Motor skill development in italian pre-school children induced by structured activities in a specific playground. PLoS ONE.

[45] Logan S.W., Robinson L.E., Wilson A., Lucas W.A. (2012). Getting the fundamentals of movement: A meta-analysis of the effectiveness of motor skill interventions in children. Child. Care Health Dev..

[46] Suttervy J., Thornton C. (2005). It doesn’t just happen! Essential contributions from playgrounds. Young Child..

[47] Little H., Wyver S. (2008). Outdoor play: Does avoiding the risk reduce the benefits. Aust. J. Teach. Educ..

[48] Sallis J.F., Saelens B.E. (2000). Assessment of physical activity by self-report: Status, limitations, and future directions. Res. Q. Exerc. Sport..

[49] McCusker K., Gunaydin S. (2015). Research using qualitative, quantitative or mixed methods and choice based on the research. Perfusion.

[50] Van Teijlingen E., Hundley V. (2002). The important of pilot studies. Nurs. Stand..

[51] Anderson T. (2005). Time Efficiency in Computer Assisted Direct Observation of Physical Activity Using SOFIT. Master’s Thesis.

[52] Crawford D., Timperio A., Giles-Corti B., Ball K., Hume C., Roberts R., Andrienopoulos N., Salmon J. (2008). Do features of public open spaces vary according to neighbourhood socio-economic status?. Health Place.

