# Supplementary material for: Physical Activity and Fundamental Motor Skill Performance of 5–10 Year Old Children in Three Different Playgrounds
Source: Int J Environ Res Public Health. 2018 Aug 31;15(9):1896. doi: 10.3390/ijerph15091896 (PMC6165260; doi:10.3390/ijerph15091896)
Supplement: Supplementary file 1 [file ijerph-15-01896-s001.pdf]

Table S1. Comparison of equipment between playgrounds.

	Traditional Playground	Contemporary Playground	Adventure Playground
Enclosed slide	✓		
Traditional slide	✓	✓	✓
Curved slide	✓		
Spiral slide	✓	✓	
Crawl tunnel	✓	✓	
Spiral	✓		
Climb wall	✓	✓	✓
Bridge	✓	✓	
Chain ladder	✓	✓	
Loop ladder	✓		
Metal ladder	✓	✓	
Steps	✓		
Fireman pole	✓	✓	
Steps	✓	✓	
Shop front	✓	✓	
Swings	✓	✓	✓
See-saw	✓	✓	✓
Wooden beam	✓		
Rockers	✓	✓	✓
Seat and metal pole	✓		
Park bench	✓		
Concrete wall	✓		
Exercise equipment	✓		
Oval/green space	✓	✓	✓
Castle		✓	
Speak tube		✓	
Mound		✓	✓
Ground slide		✓	
Rope		✓	
Poles		✓	
Tyres		✓	
Metal slide		✓	
Climb net		✓	✓
Walkway		✓	✓
Double slide		✓	✓
Uneven poles		✓	
Monkey bars		✓	✓
Flying fox		✓	✓
Swing cube		✓	
Scramble net		✓	✓
Spin roundabout		✓	
Spin poles		✓	
Climbing ropes			✓
Rock climbing wall			✓
Block steps			✓
Climbing circles			✓
Discs on poles			✓
Carved animals			✓
Wooden blocks			✓
Sandpit			✓
Chalkboard			✓
Bird nest swing			✓
Orange pipes			✓
Branches			✓
Family see-saw			✓
Spinner chair			✓
Basketball nets			✓

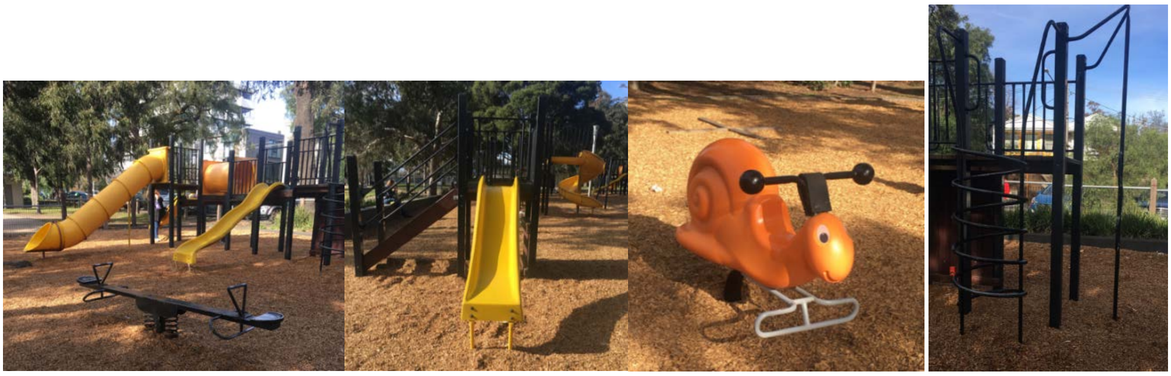

(a)

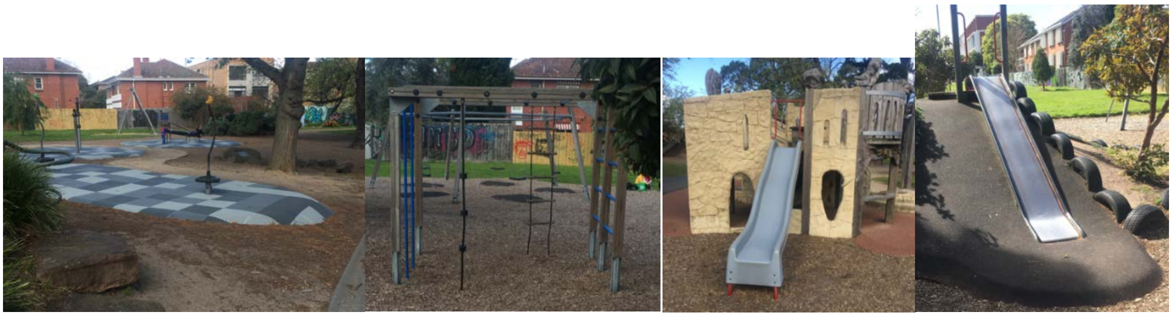

(b)

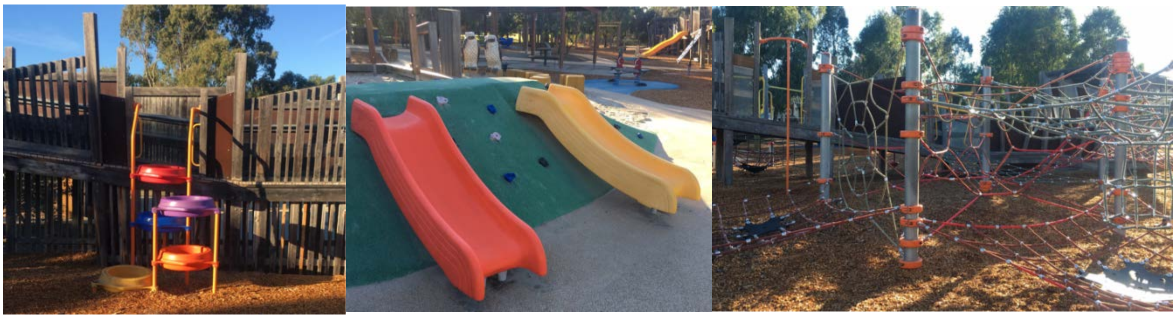

(c)

Figure S1. Images of playgrounds (**a**): Traditional playground; (**b**): Contemporary playground; (**c**): Adventure playground.

1. Stand	7. Leap	11. Balance	15. Kick
2. Walk	8. Jump	12. Climb	16. Catch
3. Run	9. Skip/Hop	13. Hang	17. Throw
4. Sit	10. Dodge/Slide	14. Pull	18. Strike
5. Lie			19. Roll
6. Crawl			

Date: ______________________________ Park: Alma Park
Start Time: _________________________ Finish Time: _________________________
Sex: ______________________________ Age: ___________________________________
ID Number: _________________________ Weather: __________________________

	Castle	Slide	Speak tube	Tunnel	Ladder	Bridge	Shop front	Steps	Mound	Steps	Ladder	Fireman pole	Ground slide	Climb Wall	Chain ladder	Rope	Ladders	Poles	Tyres	Metal slide	Climb net	Spiral slide	Fireman pole	Walkway	Double slide	Ladder	Swings	See-saw	Rockers	Uneven poles	Monkey bars	Red flying fox	Swing cube	Scramble net	Spin roundabout	Flying fox large	Spin poles	No Equipment	With Others	Alone
1																																								
2																																								
3																																								
4																																								
5																																								
6																																								
7																																								
8																																								
9																																								
10																																								
11																																								
12																																								
13																																								
14																																								
15																																								
16																																								
17																																								
18																																								
19																																								
20																																								
21																																								

Figure S2. Observation chart (Observation chart developed for the contemporary playground. For each scan, the corresponding FMS number was written in the box matching the piece of equipment the child was playing on.).
